# Effect of roughage on rumen microbiota composition in the efficient feed converter and sturdy Indian Jaffrabadi buffalo (*Bubalus bubalis*)

**DOI:** 10.1186/s12864-015-2340-4

**Published:** 2015-12-29

**Authors:** Neelam M. Nathani, Amrutlal K. Patel, Chandra Shekar Mootapally, Bhaskar Reddy, Shailesh V. Shah, Pravin M. Lunagaria, Ramesh K. Kothari, Chaitanya G. Joshi

**Affiliations:** Department of Animal Biotechnology, College of Veterinary Science & Animal Husbandry, Anand Agricultural University, Anand, Gujarat 388 001 India; Livestock Research Station, Anand Agricultural University, Anand, Gujarat 388 001 India; UGC-CAS Department of Biosciences, Saurashtra University, Rajkot, 360 005 Gujarat India

**Keywords:** Jaffrabadi rumen metagenome, roughage proportions, MG-RAST, CAZymes

## Abstract

**Background:**

The rumen microbiota functions as an effective system for conversion of dietary feed to microbial proteins and volatile fatty acids. In the present study, metagenomic approach was applied to elucidate the buffalo rumen microbiome of Jaffrabadi buffalo adapted to varied dietary treatments with the hypothesis that the microbial diversity and subsequent in the functional capacity will alter with diet change and enhance our knowledge of effect of microbe on host physiology. Eight adult animals were gradually adapted to an increasing roughage diet (4 animals each with green and dry roughage) containing 50:50 (J1), 75:25 (J2) and 100:0 (J3) roughage to concentrate proportion for 6 weeks. Metagenomic sequences of solid (fiber adherent microbiota) and liquid (fiber free microbiota) fractions obtained using Ion Torrent PGM platform were analyzed using MG-RAST server and CAZymes approach.

**Results:**

Taxonomic analysis revealed that *Bacteroidetes* was the most abundant phylum followed by *Firmicutes*, *Fibrobacter* and *Proteobacteria*. Functional analysis revealed protein (25-30 %) and carbohydrate (15-20 %) metabolism as the dominant categories. Principal component analysis demonstrated that roughage proportion, fraction of rumen and type of forage affected rumen microbiome at taxonomic as well as functional level. Rumen metabolite study revealed that rumen fluid nitrogen content reduced in high roughage diet fed animals and pathway analysis showed reduction in the genes coding enzymes involved in methanogenesis pathway. CAZyme annotation revealed the abundance of genes encoding glycoside hydrolases (GH), with the GH3 family most abundant followed by GH2 and GH13 in all samples.

**Conclusions:**

Results reveals that high roughage diet feed improved microbial protein synthesis and reduces methane emission. CAZyme analysis indicated the importance of microbiome in feed component digestion for fulfilling energy requirements of the host. The findings help determine the role of rumen microbes in plant polysaccharide breakdown and in developing strategies to maximize productivity in ruminants.

**Electronic supplementary material:**

The online version of this article (doi:10.1186/s12864-015-2340-4) contains supplementary material, which is available to authorized users.

## Background

‘Superorganism’ is the term coined to portray the association of symbiotic microbiota with its higher animal [1]. The microbes inhabiting the gut/ rumen are known to impose protective effects and nutritional benefits to the host [2] and due to its superior metabolic potentials compared to the host they are rightly considered equivalent to an organ [3–5]. In humans, microbes are found to comprise almost 3.3 million non-redundant genes, i.e. approximately 150 times higher than the number of genes in the human genome [6]. In ruminants, the significance of microbiome for maintaining host homeostasis was observed long before it was noticed in humans. Ruminant hosts are benefitted from microbial proteins and vitamins like metabolite produced by them. Ruminants obtain their energy due to the utilization of complex plant polysaccharides by microorganism, which are else indigestible by host. Domestication of ruminants has provided stable food supply and means of livelihood to thousands of people worldwide since earlier times. Ruminant meat and milk is considered as one of the most important agricultural products (FAO, 2009). Because of concomitant increase in food consumption, the demand for meat and milk is constantly increasing, further enhancing the need for sustainable number of ruminants (World Bank, 2008). Sustainable production of meat and milk requires an in-depth knowledge about the functions of the microbes and their interactions with the host. Nowadays, the high-throughput next generation sequencing technologies have alleviated studies such as metagenomics, metatranscriptomics, metaproteomics and metabolomics to characterize complex rumen microbial ecosystem and apply the information for improving forage digestion efficiency.

On the other hand, the feed composition also profoundly influences the microbial population [7, 8]. Ruminants possess a very complex microbial ecosystem comprising of bacteria, protozoa, fungi, methanogens and bacteriophages that well adapt to variable diets [7, 9]. Their abundance in the rumen and the metabolic activity is reported to be remarkably high compared to terrestrial and aquatic ecosystems [10–13]. However, very few (<15 %) of the rumen bacteria are known to be cultured [14], highlighting the importance of molecular biology approaches like metagenomics to sidestep this limitation.

Ruminants are a crucial intermediate between light energy and the production of edible compounds such as milk and meat. Among them, buffalo plays a highly vital part of the Indian economy solely contributing for >55 % of total milk production by Indian livestock. Especially, the Jaffrabadi breed of buffaloes, which are mainly a native of Saurashtra region including the Junagadh, Bhavnagar, Jamnagar, Porbandar, Amreli and Rajkot districts of Gujarat are good milkers and one of the heaviest Indian breed. They efficiently convert roughage into milk and are found to be different compared to other breeds in the terms of its milk fat content [15]. Earlier reports have described use of rumen microbiota modulation for increasing the feed digestion efficiency [16, 17]. Ruminants can degrade dietary fibers to short chain fatty acids with help of the inhabiting microbial consortium. In the process, ATP is generated that stimulates microbial growth, specifically the synthesis of cellular protein [18]. This can be an important attribution for improving the energy supply of rumen. Maximizing efficiency of its production would consequently improve cattle productivity. Nevertheless, knowledge of the functioning of rumen microbiome for example mechanism of plant polysaccharide degradation is still incomplete [19, 20]. The evolution of the study of rumen microbial diversity similar to other microbial ecosystems has moved from culture-based microscopic studies to the use of culture-independent molecular techniques. Second-generation sequencing techniques have revolutionized the microbiome study by producing huge amount of data leading to increased coverage depth further permitting identification of even the less abundant community members [21, 22]. Phylogenetically near microbes are reported to have varied metabolic potentials thus revealing the significance of characterizing complex microbial communities [23, 24]. Rumen microbiome studies of Indian cattle and buffalo is gaining much interest, however the Jaffrabadi breed rumen microbiome has not been well characterized. The objective of this study was to characterize and compare the rumen microbiome at taxonomic and metabolic level as well as to establish the database of the same for efficient feed converter and sturdy Indian breed of buffalo, Jaffrabadi (*Bubalus bubalis*) reared under varied dietary roughage proportions using metagenomic approach to better comprehend its correlation with nutrient utilization and maintenance of host homeostasis.

## Methods

### Ethics Statement

The experiment was performed under the approval of the institutional animal ethics committee of College of Veterinary Science & Animal Husbandry, Anand Agricultural University vide letter no. AAU/GVC/CPCSEA-IAEC/108/2013 dated 05/10/2013.

### Dietary Feed treatment

In the present study, eight healthy adult (4–5 years old), non-pregnant, dry buffaloes of Jaffrabadi breed which had completed 1^st^ lactation and had an average body weight of 350–400 kg were included. All animals under study were reared at the Livestock Research Station, Anand Agricultural University, Gujarat, India. Before the experimental feed treatment, the animals were maintained under the feeding standards of the National Royal Commission (NRC). Out of eight animals under study, four were supplemented with green (G) fodder as roughage content and another four with dry (D) fodder as roughage content in total feed (roughage and concentrate). The nutrient composition of the feed were described in Additional file 1: Table S1. Considering the facts that if started with dietary treatment of 100 % roughage (D/G), the microbial diversity lost due to low protein supplement may take a long time to recover when shifted to high protein diet, whereas if started with a 50 % of roughage (D/G), the available microbial diversity will start to adapt the less protein diet. Thus, in this study feeding regime included an increasing proportion of roughage (D/G) content at regular feed intervals. A total of three dietary treatments in the current study comprised a mixture of roughage(R) and concentrate (C) feed at ratios of 50:50 (J1),75:25 (J2) and 100:0 (J3). Each dietary treatment was continued for six weeks to permit microbial adhesion and adaptation to the same, the time duration also ensures less probability of the previous microbial diversity carry over effects. After six weeks, rumen sample was collected from animals using stomach tube and vacuum pump 3 h post feeding. The rest of the rumen fluid was then passed through muslin cloth, without pressure for separation of the liquid (L) and solid (S) fractions without any wash. The filtrate and the solid fraction were collected in sterile cryo vials and were stored at −80 °C until metagenomic DNA extraction. Simultaneously, prior to filtration, a small proportion of the rumen fluid (~100 ml) was aliquoted in a bottle for total volatile fatty acid (TVFA) and ammonia estimation. TVFA was measured using the Markham's distillation apparatus, wherein the volatilized fatty acids in presence of oxalate was collected through condenser and calculated, and the nitrogen contents were measured by the Kjeldahl's method.

### Metagenomic DNA extraction

Metagenomic DNA was isolated from liquid and solid rumen samples of all the three treatments (4 animals x 2 roughage types x 3 diet treatments x 2 fractions: total 48 samples) using QIAmp DNA Stool Mini Kit (Qiagen, USA) with slight modification for the solid samples that included pretreatment of PBS buffer along with vortexing for an hour to release the bacteria adhered to the dietary fibers and minimize the plant DNA fraction during the extraction procedure. DNA quality and quantity was determined using a NanoDrop ND-1000 spectrophotometer (Thermo Fisher, USA) and on 0.8 % agarose gel electrophoresis.

### Shotgun Sequencing of rumen microbiome

Forty eight DNA shotgun bar-coded libraries from a start-up DNA concentration of ≥300 ng of each sample were prepared as per standard 400 bp Ion Torrent PGM protocol (Life Technologies, USA). Four barcoded libraries of biological replicates from all the treatments were pooled on an equimolar basis. Emulsion PCR was carried out using the Ion OneTouch™ 400 Template Kit followed by enrichment of the Ion Sphere Particles (ISPs) as per the standard protocol. The enriched ISPs were loaded on the 316 chip, and sequencing run was performed. Twelve sequencing runs (4 barcoded libraries per run) were carried out on Ion Torrent PGM.

### Taxonomic and Functional analysis

All metagenomic reads were uploaded on MG-RAST (Metagenomic Rapid Annotations using Subsystems Technology v3.2) for annotation purpose (http://metagenomics.anl.gov/) [25]. Host-specific reads were curtailed by screening the reads against the closest relative *Bos taurus* UMD v3.0, as published complete sequences for buffalo were unavailable in the database. Phylogenetic classification was performed against M5NR database with minimum e-value of 1e-5 and minimum identity of 60 %. Each value obtained indicated the percentage/percent relative frequency of reads with predicted proteins and ribosomal RNA genes annotated to the indicated taxonomic level. Functional annotation was performed using the SEED database with a minimum e-value of 1e-5 and minimum identity of 80 % at level 1 and level 2. For all subsequent analysis, mean proportion of four animals maintained under same diet treatment were considered, unless mentioned otherwise.

### CAZymes mining from rumen metagenome

The raw reads from four replicates were pooled and assembled into contigs using GS De Novo Assembler v2.3 with default parameters. The assembled contigs were uploaded in CAZymes Annotation Toolkit (CAT) at http://mothra.ornl.gov/cgi-bin/cat/cat.cgi for sequence based annotation with e-value of 1e-5 [26]. The results were further studied for recognizing the organisms contributing to coding sequences (CDS) for CAZymes.

### Statistical analysis

Two-way Fisher’s exact test with a Benjamin-Hochberg FDR multiple test correction was performed for statistical analysis between all the rumen microbiome samples at the taxonomic and functional level using the Statistical Analysis of Metagenomic Profiles, STAMP v1.07 software package for identification of biologically meaningful statistical differences between samples at different variable including treatment, fractions and diet [27]. Metabolism and CAZyme abundance were analyzed for statistical significance based on correlation test and ANOSIM (Analysis of Similarity) using PAST (Paleontology Statistics) software [28].

### Nucleotide sequence accession numbers of rumen Microbiome samples

All the sequences were submitted to MG-RAST server for annotation (Details in Table 1).

**Table 1:** Sequence details of 48 metagenomic samples

Sample ID	MG-RAST Accession No.	Initial Reads	QC Passed Reads	rRNA Reads	Reads with predicted CDS	Post QC bp Count	α Diversity
J1GL1	4604206.3	1,424,078	1,173,142	1,066	1,005,811	229 MB	278.47
J2GL2	4604108.3	928,450	742,323	638	619,505	127 MB	322.03
J1GL3	4604092.3	512,016	423,203	390	368,554	77 MB	330.86
J1GL4	4571939.3	412,187	336,762	1,541	283,379	52 MB	422.45
J1GS1	4604096.3	498,599	412,088	535	353,673	74 MB	305.85
J1GS2	4604097.3	549,260	465,291	815	414,950	93 MB	226.21
J1GS3	4604098.3	677,970	562,220	820	494,011	111 MB	267.64
J1GS4	4604100.3	703,240	571,552	624	490,396	105 mb	293.22
J1DL1	4575792.3	949,870	636,956	5,020	516,485	106 MB	236.28
J1DL2	4575793.3	774,918	559,938	4,449	454,942	96 MB	215
J1DL3	4575794.3	918,259	673,358	5,722	546,797	115 MB	192.89
J1DL4	4575795.3	845,121	589,649	5,080	480,019	98 MB	238.12
J1DS1	4575901.3	708,081	451,811	2,716	355,948	72 MB	295.41
J1DS2	4575796.3	951,833	680,197	4,127	549,267	115 MB	309.42
J1DS3	4575797.3	641,328	463,939	2,576	386,658	79 MB	265.89
J1DS4	4575798.3	700,602	505,592	3,066	414,480	87 MB	271.48
J2GL1	4575799.3	505,950	311,420	1,779	250,659	47 MB	246.79
J2GL2	4575800.3	622,310	363,544	2,924	283,594	52 MB	272.56
J2GL3	4575801.3	1,029,049	580,314	3,600	449,782	84 MB	216.72
J2GL4	4575802.3	752,725	428,414	2,834	330,438	62 MB	249.28
J2GS1	4575803.3	632,004	364,726	2,382	287,106	53 MB	198.09
J2GS2	4575804.3	723,939	405,675	2,573	315,656	59 MB	187.26
J2GS3	4575805.3	672,315	386,374	2,586	302,656	56 MB	188.61
J2GS4	4575806.3	625,425	359,933	2,566	278,956	53 MB	179.03
J2DL1	4575807.3	1,051,264	753,416	10,146	631,726	148 MB	282.88
J2DL2	4603329.3	713,045	597,496	678	528,928	145 MB	343
J2DL3	4575809.3	986,698	722,822	9,061	609,515	144 MB	210.54
J2DL4	4575810.3	850,672	603,721	8,710	504,032	116 MB	320.17
J2DS1	4575811.3	840,552	588,345	9,514	473,773	108 MB	257.68
J2DS2	4575812.3	477,350	295,723	5,475	231,448	48 MB	314.28
J2DS3	4575813.3	615,868	389,436	7,503	302,586	62 MB	330.97
J2DS4	4618633.3	659,273	522,750	506	450,497	111 MB	347.89
J3GL1	4604101.3	652,383	552,242	603	490,170	55 MB	324.08
J3GL2	4575822.3	1,110,839	796,087	8,867	621,768	140 MB	302.69
J3GL3	4575823.3	635,610	456,220	3,733	376,693	82 MB	261.99
J3GL4	4575824.3	730,149	533,490	3,910	439,428	97 MB	296.69
J3GS1	4575825.3	626,583	392,629	3,997	313,489	66 MB	244.66
J3GS2	4575826.3	669,967	456,754	3,937	375,022	81 MB	239.68
J3GS3	4575902.3	774,116	497,294	5,187	391,485	85 MB	276.19
J3GS4	4575897.3	715,753	470,224	4,152	385,107	83 MB	257.78
J3DL1	4575814.3	886,334	609,043	6,891	504,556	107 MB	332.67
J3DL2	4575815.3	703,305	512,015	5,212	437,145	90 MB	399.25
J3DL3	4575816.3	703,848	511,130	5,656	432,451	89 MB	386.36
J3DL4	4575817.3	1,072,487	735,953	7,679	607,004	124 MB	373.05
J3DS1	4604102.3	3,651,797	2,657,826	2,473	1,941,947	530 MB	217.07
J3DS2	4575818.3	503,637	309,631	4,059	236,682	47 MB	171.43
J3DS3	4575819.3	512,538	334,694	4,934	266,702	54 MB	218.68
J3DS4	4575820.3	762,281	443,019	8,521	328,072	60 MB	318.59

## Results

Metagenome sequencing of 48 samples using Ion torrent resulted into a total of 38 million raw reads and 27 million reads post quality filtering. The sequencing data of individual samples obtained after quality filtering are detailed in Table 1. Based on the MGRAST annotation, about 0.1 to 1.9 % of the reads were classified as rRNA per rumen microbiome sample based on the hits against 16S rRNA gene sequence databases while 73 to 90 % of the reads from all the samples were classified into various functional subsystems.

### Taxonomic distribution in the rumen microbiome samples

At the domain level, bacteria were the most abundant with an abundance of >90 % in all the samples, followed by the eukaryotes (~3 to 5 %). Archaea were present in a proportion of ~1 %, while the rest included unassigned sequences and the virus sequences.

### Effect of diet on microbial diversity

**Fig. 1:**
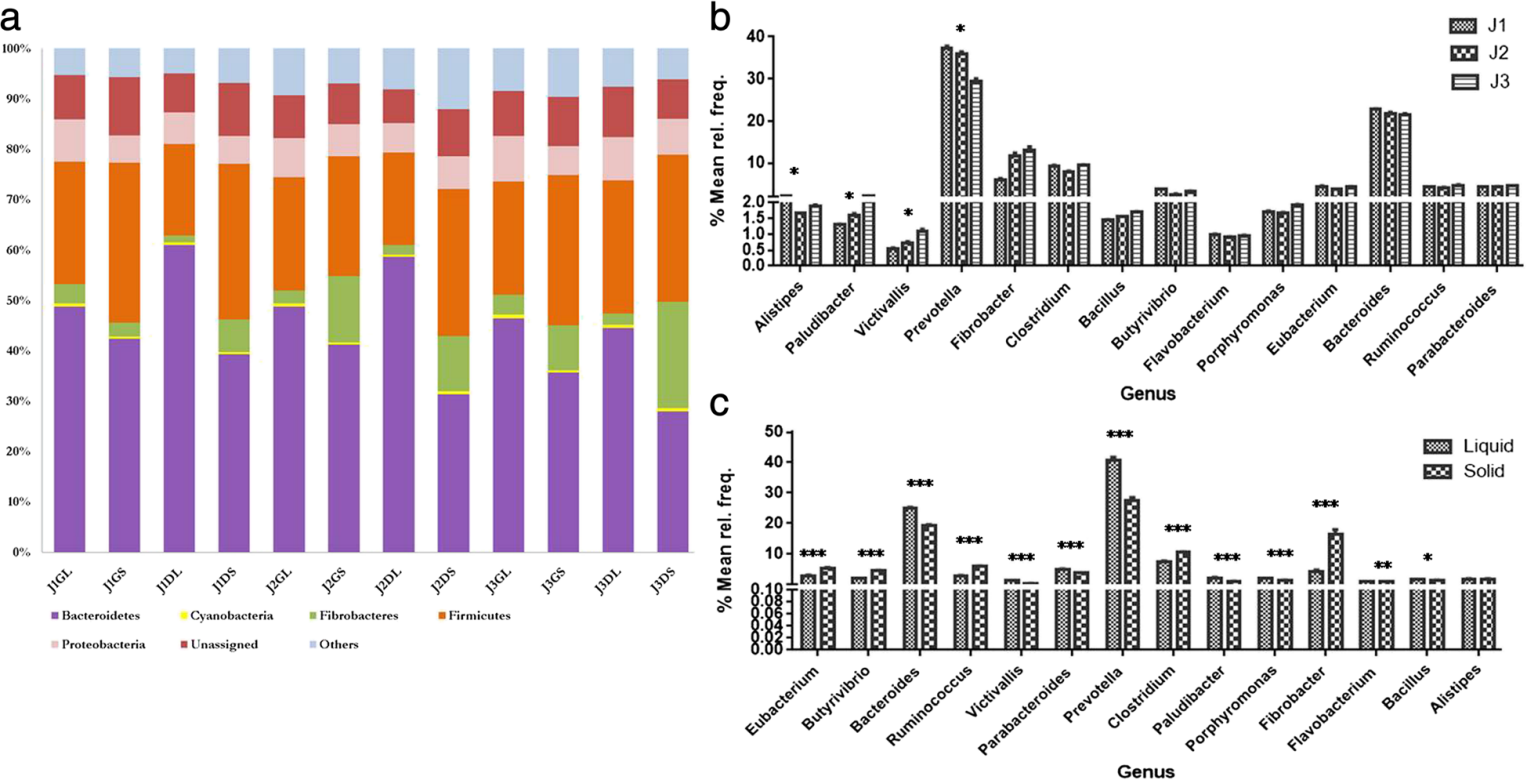
**a** Taxonomic distribution of bacterial phyla based on predicted proteins and rRNA in the samples [Treatment vs Abundance in percentage] **b** Statistical analysis using STAMP based on genus level taxonomic assignments between treatments **c** Statistical analysis using STAMP based on genus level taxonomic assignments between liquid and solid fraction (*indicates *p* < 0.05, **indicate *p* < 0.01, ***indicate *p* < 0.001)

The taxonomic assignment at phylum level using the M5NR database revealed dominance of the *Bacteroidetes* phyla in the rumen microbial community of liquid fraction of Green Roughage (GL) as well as the Dry Roughage (DL) diet fed animals (Fig. 1a). *Firmicutes, Fibrobacter, Proteobacteria* and *Cyanobacteria* were the other major phyla observed constituting about 80 – 85 % of the total phyla across all the three, i.e. J1, J2 and J3 treatments. Rest 15 to 20 % comprised of majorly unassigned sequences followed by other phyla including *Actinobacteria, Chlamydiae, Spirochaetes, Tenericutes* and *Verrucomicrobia. Bacteroidetes* were observed to be lower in the solid samples as compared to that in liquid samples, whereas the *Fibrobacter* and *Firmicutes* proportion was relatively higher. As the roughage proportion was increased from 50 % to 100 % there was decrease in the *Bacteroidetes* abundance across all the samples.

Similarly, at the genus level the two predominant genus of *Bacteroidetes* family, viz. *Prevotella* and *Bacteroides* were found decreasing with the increase in roughage proportion (Fig. 1b). Both were significantly (p-value < 0.05) higher in the liquid as compared to solid portion (Fig. 1c). Out of the fourteen most abundant genera comprising about 70 % of total genera, four were significantly varying across treatments and thirteen were varying between the solid and liquid fractions. *Paludibacter* and *Victivallis* were found increasing with the increase in roughage content. *Allistepes* was highest in J1, followed by J3 and then J2. Further at species level, *Prevotella ruminicola* was the predominant species in all the liquid samples throughout different treatments followed by the two important fibrolytic species of rumen ecosystem viz., *Fibrobacter succinogenes* and *Ruminicoccus albus.* In the solid fraction, *Fibrobacter succinogenes* and *Ruminicoccus albus* were predominant followed by *Prevotella ruminicola*.

### Roughage vs. *Firmicutes/Bacteroidetes* ratio

**Fig. 2:**
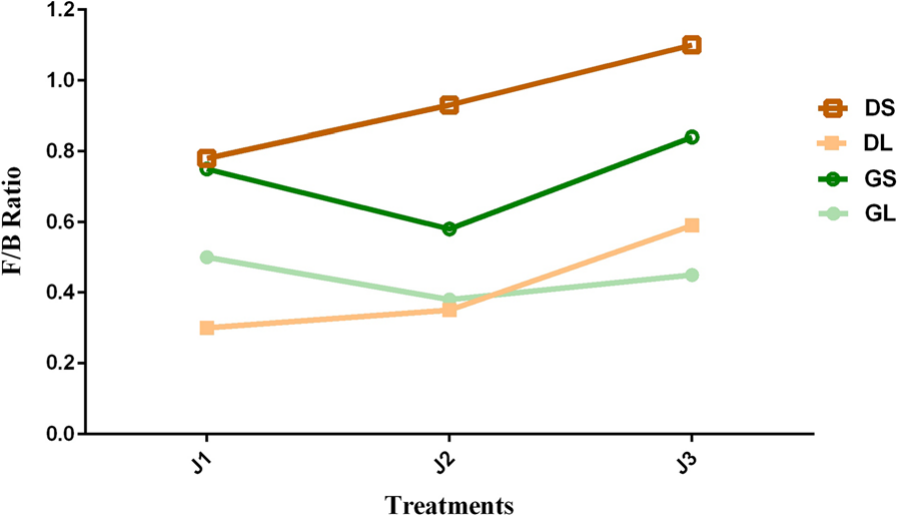
*Firmicutes/Bacteroidetes* ratio during the three treatments

*Bacteroidetes* and *Firmicutes* were the most prevalent phyla throughout the treatments. For the *Firmicutes*/*Bacteroidetes* ratio, significant differences between liquid (0.3 to 0.59) and solid (0.6 to 1.1); as well as between the three treatments were observed for both green and dry roughage fed animals (Fig. 2). Notably specific trend was observed for green and dry diets, whereby in the green roughage fed animals the ratio was found to be higher in J1, reduced in J2 and again increased in the J3 treatment, whereas in dry roughage fed animals the ratio was found increasing with increase in roughage proportion (J1 > J2 > J3).

### Diet and Correlation between various Phyla

We checked for the cross-phylum interactions in the buffalo rumen among green and dry diet feeds. Phylum abundance profiles were determined across 12 rumen samples. Linear correlation test revealed negative correlation was observed between the *Bacteroidetes* and *Fibrobacters* phyla. During green roughage treatment results showed that the abundance of *Bacteroidetes* positively correlated with abundance of bacterial phylum *Verrucomicrobiae*, *Proteobacteria* and *Tennericutes* for both the treatments while negatively correlated with *Fibrobacters, Firmicutes* and *Spirochaetes* during the dry roughage treatment (Table 2)*.*

**Table 2:** Correlation between various phyla using PAleontologicalSTatistical analysis among Green Roughage fed animals

Dry	Bacteroidetes	Fibrobacter	Firmicutes	Proteobacteria	Spirochaetes	Tenericutes	Verrucomicrobia
Green							
Bacteroidetes	--	−0.876**	−0.437	0.369	−0.552	0.386	0.429
Fibrobacter	−0.569	--	0.586	−0.378	0.727	−0.477	−0.519
Firmicutes	0.404	0.022	--	0.318	0.772*	0.151	0.165
Proteobacteria	0.897*	−0.258	0.244	--	0.334	0.945***	0.978***
Spirochaetes	0.661	0.087	0.591	0.810	--	0.162	0.165
Tenericutes	0.865*	−0.540	−0.047	0.912**	0.568	--	0.966***
Verrucomicrobia	0.809	−0.315	−0.039	0.932	0.647	0.953**	--

Note: *indicates p < 0.05, **indicate p < 0.01, ***indicate p < 0.001

### Functional diversity based on dietary changes

**Fig. 3:**
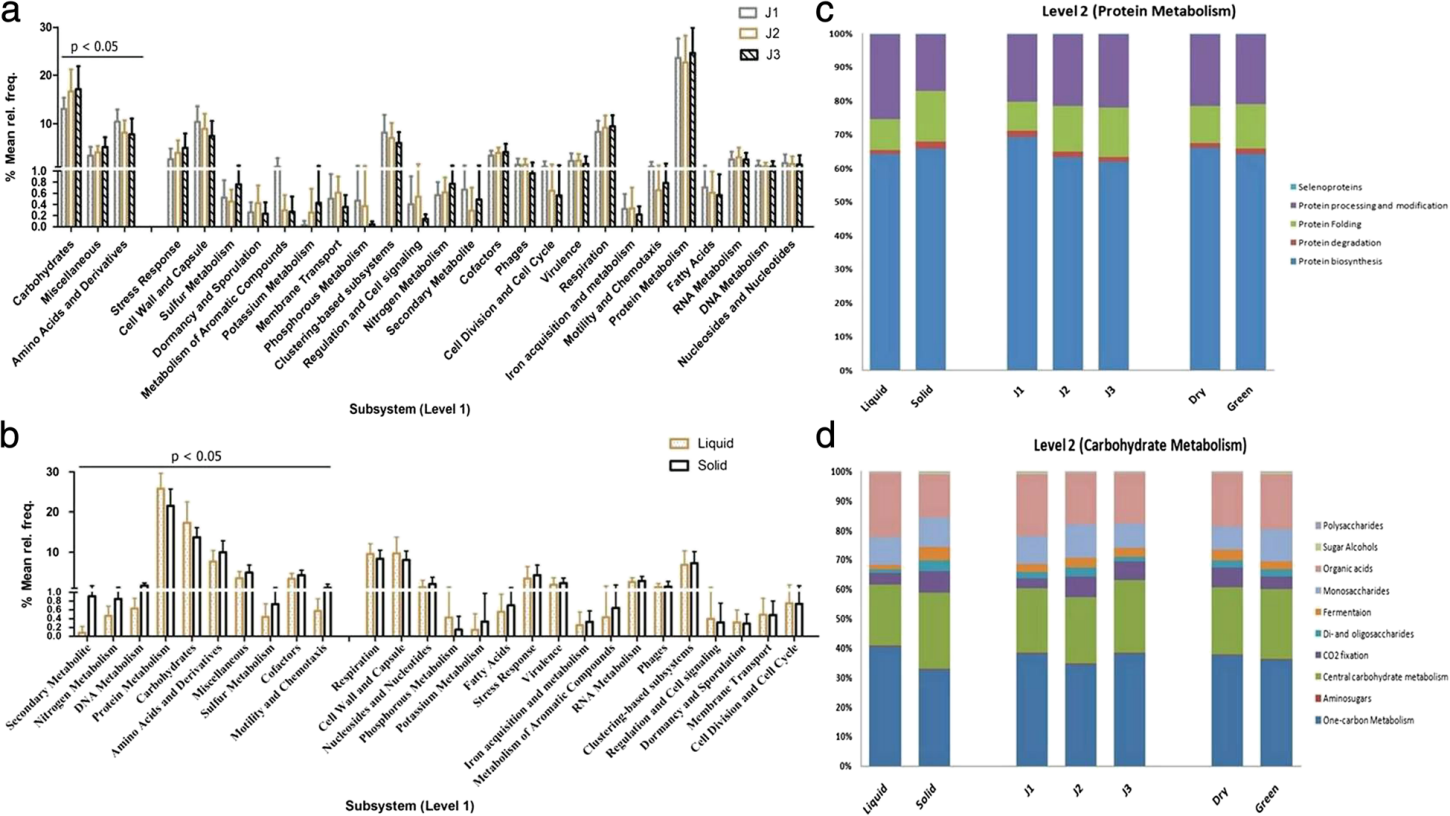
**a** Statistical analysis using STAMP based on functional assignments at subsystem level 1 between treatments (*p* < 0.05) **b** Statistical analysis using STAMP based on functional assignments at subsystem level 1 between treatments (*p* < 0.05) between liquid and solid fraction (*p* < 0.05) **c** Distribution of Protein Metabolism subsystem at level 2 between treatments, diet and fractions [Sample type vs Abundance of categories in percentage] **d** Distribution of Carbohydrate Metabolism subsystem at level 2 between treatments, diet and fractions Metabolism [Sample type vs Abundance of categories in percentage]

Functional analysis at level 1 using the SEED database in MG-RAST revealed that the highest coding sequences were present for the protein metabolism followed by carbohydrate metabolism category. At level 1, about ten categories including secondary metabolite; nitrogen metabolism; DNA metabolism; protein metabolism; carbohydrates; amino acids and derivatives; sulfur metabolism; cofactors, motility and Chemotaxis; and miscellaneous were observed to be significantly different between the liquid and solid fractions (Fig. 3a). Among these, the abundance of all categories was more in the solid fraction except the carbohydrate and protein metabolism which was higher in the liquid portion, which clearly indicated the difference in the microbial diversity of both the fractions. Three of the categories viz. carbohydrates; amino acids and its derivatives; and miscellaneous differed significantly across the J1, J2 and J3 dietary treatments, wherein the coding sequences related to the Carbohydrate subsystem increased with the increase in roughage concentration (Fig. 3b).

Further based on the highest abundance of protein and carbohydrate metabolism, we studied the level 2 classification for these two subsystems. Protein metabolism included five different categories including protein biosynthesis, protein degradation, protein folding, protein modification and processing and selenoproteins (Fig. 3c). The abundance of all the categories except selenoproteins varied across the three treatments; green and dry diets as well as the liquid and solid fractions. We observed that the maximum sequences were attributed to the protein biosynthesis, followed by protein modification and processing. With increase in the roughage proportion, protein biosynthesis and degradation were found to be reducing while protein modification, processing and refolding were increasing. During the dry diet, protein biosynthesis was more abundant, while the degradation and folding were more expressed in green diet. All the categories except protein processing and modification were more expressed in the solid fraction as compared to the liquid fraction.

Similarly, the carbohydrate metabolism included ten sub categories, among which the central carbohydrate metabolism, amino sugars and polysaccharides metabolism did not vary across the samples (Fig. 3d). One carbon metabolism, organic acids and sugar alcohol were least in the J2 treatment compared to J1 and J3. While monosaccharide and oligosaccharides metabolism, fermentation and CO_2_ fixation was highest in the J2 treatment. All the categories were more abundant in the dry treatment compared to the green treatment. Among the two fractions, solid fraction gave more hits to the functional category as compared to the liquid fractions.

**Fig. 4:**
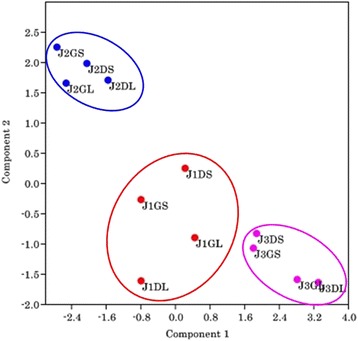
Principal component analysis based on taxonomic (phylum level) and functional distribution in each sample using PAST

Further, to understand the similarity/variation among the microbiome composition of all the samples, principal components analysis (PCA) was performed for between groups correlation based on taxonomic and functional annotation. Principal component analysis (PCA) of showed that the samples fed on same treatment (J1, J2 and J3) clustered together (Fig. 4). Samples from each treatment of green and dry roughage fed animals tended to cluster together, signifying that related functions were encoded in their metagenome during respective treatments.

### Diet impact on Rumen Metabolism

In our study, to determine the effect of change in feed on rumen metabolism, we performed whole rumen fluid analysis. The total-N and ammonia-N concentrations in the rumen fluid were found decreasing significantly (*p* < 0.05) in the dry roughage fed animals with increase in the roughage proportion viz. from J1D to J3D. Also, the concentrations of total–N and ammonia-N were relatively higher in the green roughage fed individuals as compared to the dry roughage fed individuals. Soluble N was significantly higher in J1 as compared to J2. The concentration of Non Protein Nitrogen (NPN) was found to be significantly higher in green roughage fed individuals as compared to dry roughage fed. The concentration of NPN also significantly decreased with increase in the roughage proportion (J1 > J2 > J3) for both green as well as dry roughage fed individuals. The Volatile Fatty Acid (VFA) concentrations did not show significant variation, though they were observed to be relatively higher in J1 and J3 compared to J2, but the difference was statistically non-significant (Table 3).

**Table 3:** Analysis of variance showing the effect of diet on various metabolites

Dietary Treatment		J1(50R:50C)		J2(75R:25C)		J3 (100R:0C)	
Metabolite		Dry	Green	Dry	Green	Dry	Green
Nitrogen Content (mg/dL)	Total N	77.7^aA^ ± 5.8	147.7^b^ ± 14.01	63.49^B^ ± 6.63	64.75 ± 6.12	25.55^cC^ ± 1.55	48.55^d^ ± 9.36
	Ammonia N	8.22^aA^ ± 0.25	20.75^b^ ± 1.05	7.21^B^ ± 1.23	10.61 ± 1.84	3.08^aC^ ± 0.37	11.62^b^ ± 1.12
	Soluble N	21.98^A^ ± 0.54	37.24^C^ ± 1.39	14.42^B^ ± 1.38	14.07^D^ ± 0.83	11.26 ± 0.49	12.67 ± 0.7
	NPN	42.42^aA^ ± 0.77	53.2^bD^ ± 2.93	25.2^cB^ ± 2.54	29.12^dE^ ± 2.71	14.7^eC^ ± 1.24	23.1^fF^ ± 1.2
Total VFA (mM/L)		4.48 ± 0.51	4.67 ± 0.29	4.34 ± 0.4	4.55 ± 0.72	5.33 ± 0.3	5.5 ± 0.17

Mean values in the same row with different superscripts (Capital case for between treatment; small case for between Dry/Green) differed significantly (*P* < 0.05)

### Dietary effect on volatile fatty acid (VFA) production

Environmental gene tags (EGTs) involved in production of volatile fatty acids viz. propanoate and butanoate were studied to check the effect of different diets on the same as (Pathway illustrated in Additional file 2: Figure S1). The results showed that the EGTs for propanoate were abundant in the first treatment and nearly likewise represented during the second and third treatment. Results also revealed that the propanoate production via 2-hydroxy butyrate was better represented as compared to the Succinate pathway.

Similarly, EGTs corresponding to the butanoate production pathway were also determined throughout the dietary treatments. The biosynthesis pathway for butanoate production involves the final step for butanoate formation from butanoyl CoA via two possible pathways (Additional file 3: Figure S2). The results showed that the EGTs for butanoate were abundant in the J1 treatment subsequently followed by J3 and J2 treatments, except the EGTs for phosphate butyryl transferase and butyrate kinase enzymes responsible for the final butanoate production which were comparatively more abundant in the J2 than the J3 treatment.

### Dietary effect on Methanogenesis pathway

We also considered the effect of three different dietary treatments on the proportion of EGTs involved in methane production (Additional file 4: Figure S3). The entire anaerobic process of methane formation takes place by the methanogenic archaea population in the rumen. EGTs for enzymes involved in methanogenesis were abundant in J1 followed by J2 and J3 treatment. Thus, increase of roughage proportion in the diet reduces the active genes for production of methane.

### Diet based variation in CAZymes distribution

Results of the CAZyme Analysis Tool (CAT) revealed that the most encoded CAZyme domain included the Glycoside Hydrolase (GH) family (~50 % of total assignment), followed by the Carbohydrate Binding Modules (CBM) (~30 %), Glycosyl Transferases (GT) (~15 %) and a rest of the proportion (~5 %) comprising Pectate Lyases (PL) and Auxiliary Activities (AA) (Additional file 5: Figure S4). The proportion of GH family was found to increase marginally with increase in the roughage proportion for the dry fed individuals. GT family was represented more in the liquid samples as compared to the solid samples.

**Fig. 5:**
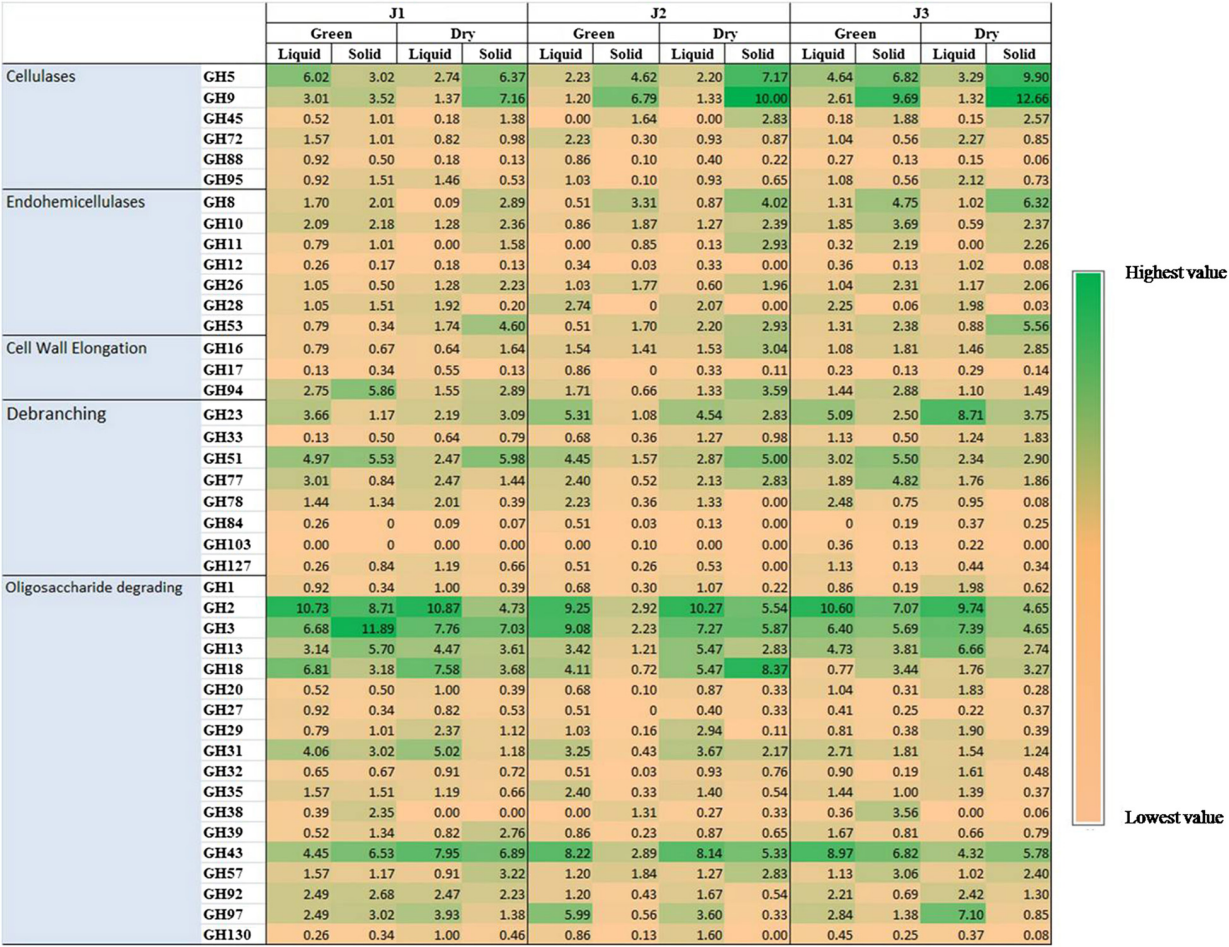
Glycosyl Hydrolase family distribution (Pooled de novo assembly of 4 animals maintained under same diet treatment was considered)

GH family can be further classified based on the functional role of the enzymes such as degradation of oligosaccharide, cellulose, hemicelluloses, pectin and other plant polymers. The contigs were found to comprise sequences related to a total of 52 different GH families amongst which majority included those playing role as oligosaccharide degrading enzymes (18 families) followed by debranching enzymes (8 families), cellulose degrading endohemicellulases (7 families) and cellulases (5 families), and cell wall elongation related enzymes (3 families) (Fig. 5). Out of these, oligosaccharide degrading GH2, GH3, GH18, and cellulase families GH5 and GH9 were most highly represented. The abundance of the GH families encoding gene varied with respect to the treatments; green and dry diet; as well as the solid and liquid fractions. Among the cellulases, GH5 and GH9 were the most abundant and were found to be more in the solid phase as compared to the liquid phase irrespective of diet, except for the GH5 in J1 treatment wherein it was more abundant in liquid compared to the solid phase. For the liquid phase the abundance of the former two families was more in the green diet fed individuals as compared to the dry fed. While for the solids the trend was found to be different viz. less in green compared to dry. Increasing the roughage proportion increased the cellulase encoding EGTs in the solid phase of both green and dry diet fed individuals, whereas for the liquid the abundance was higher in J1, reduced in J2 and again increased in J3. Similarly the endohemicellulases were found to be more abundant in the solid phases as compared to liquid, except for the GH12 and GH28 families which were almost negligible in the solid samples, with increase in the roughage (i.e. during the J2 and J3 treatments). Majority of the GH families in oligosaccharide degradation were more abundant in the liquid and well represented in the J1 and J3 treatments as compared to the J2 treatment except GH3, GH43, GH35, GH39, GH97 and GH130 which had slightly higher proportion in J2GL. The results thus reflect that diet has an impact on the microbial diversity further resulting in variability of the CAZyme families present in the studied samples.

We studied the taxonomy of the organism coding the GH families at genus level.

**Fig. 6:**
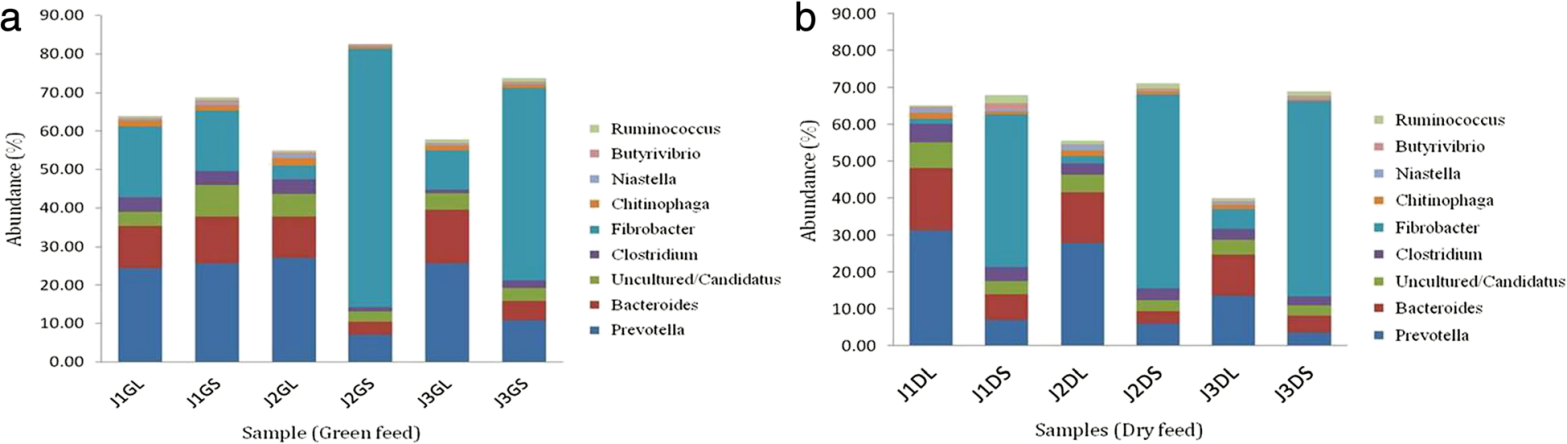
Taxonomic distribution at genus level of microbes encoding genes for predicted CAZyme [Treatment vs Abundance in percentage] (**a**) Green roughage fed animals (**b**) Dry roughage fed animals

While in the green roughage fed individuals, there was enrichment of major microbial communities involved in encoding GH during the 75 % roughage diet for liquid fraction, except for *Bacteroides* that were least during the J2DL treatment (Fig. 6a). In the solid samples, *Fibrobacter* were most abundant in J2, while *Prevotella*, *Bacteroides* and *Clostridium* were least. Hence, based on the results it is evident that *Prevotella* and *Fibrobacters* genus is the most active in terms of genes encoding CAZymes and hence can be exploited for industrial purposes.

In the dry roughage fed individuals, it was clearly observed that *Prevotella* was dominant in the liquid fractions and *Fibrobacter* in the solid (Fig. 6b). Also with increasing roughage the GH encoded by *Prevotella* reduced for both solid and liquid. Also in the liquid samples, the *Ruminicoccus* genus was observed much higher in J2 and J3 as compared J1, whereas in solid it was found to be more in J1 and then reduced in J2 and J3. The *Fibrobacter* genus was found to be coding more enzymes with increased roughage diet. The *Chitinophaga* genus was abundant in J2 treatment and *Clostridium* in J1 treatment for both the fractions. *Bacteroides* genus was less in the J2DS samples as compared to J1DS and J3DS, while it was highest in J1DL followed by J2DL and then J3DL.

## Discussion

Ruminants entirely depend on their microbial community for feed digestion. Hence, appealing a hypothesis that there exists a strong correlation between the microbes and the host physiology focusing on nutrition availability and development. Understanding of the rumen microbiome has comprehensive implications like effect on methane production by rumen methanogens to host health maintenance and animal production [29–31]. Rumen microbiome of the sturdy Jaffrabadi breed buffalo native of the western India has not been deeply characterized as yet, especially by using the metagenomic approach.

In context to the same, present study focused exploration of diet dependent variation in the rumen bacterial community composition and functional characterization of 48 rumen samples of Jaffrabadi breed. Four animals as biological replicates were considered to evaluate effect of different proportions of green and dry roughage so that any considerable animal to animal microbiome variation can also be observed. However, as previously reported we observed lower variation between replicates [32]. We analyzed the effect of increased roughage proportion in diet on fiber adhered and fiber free microbial populations and found that particular taxa are enriched/suppressed with the change in diet and the functional abilities related to fulfilling host energy requirements are correlated with the abundance of various bacterial taxa.

### Effect of diet on microbial diversity

*Bacteroidetes, Firmicutes, Fibrobacter* and *Proteobacteria* were the four dominant phyla observed in the present study. The results were similar to several of the earlier mammalian gut/rumen microbiota studies [33–36]. The *Bacteroidetes* were the most abundant phyla in majority samples, while few of the solid fraction samples had dominance of *Firmicutes*, similar to the earlier results observed in bovine rumen [37, 38]. Reports have also revealed that the increase of fibrous content in diet increased the *Firmicutes* abundance and decreased the *Bacteroidetes* population in the gut microbiota, as also observed in our results [37, 39]. The abundance was evident at the genus level also, where the *Prevotella* and *Bacteroides* genus dominated. *Bacteroides* group depend on the soluble polysaccharides released in liquid phase of rumen by the activities of other bacteria [40, 41]. *Prevotella* have been shown to be involved in volatile fatty acid production like propanoate further used for energy by the host [42]. Increased *Prevotella* abundance could also be a reason in reducing methanogenesis as reported in a companion experiment [30, 43]. Along with *Bacteroidetes, Firmicutes* was one of the most abundant phyla in the rumen. At genus level, *Clostridium, Ruminococcus* and *Butyrivibrio* were the most represented *Firmicutes*. They are potent cellulose degraders as well as have the ability to digest pectin and hence are important component in the rumen dietary fiber utilization [44]. *Bacteroidetes* and *Firmicutes* population in the rumen and gut collectively involve in the conversion of organic matter to simpler forms [45, 46]. The solid fraction favored *Fibrobacteres,* suggesting their importance in fiber degradation similar to the results of microbiome analysis of cattle fed Pasteur diets. However the results were contradictory to those reported earlier in mammalian guts, where the abundance was more in liquid fractions [34, 37].

### Roughage vs. *Firmicutes/Bacteroidetes* ratio

The *Firmicutes* to *Bacteroidetes* ratio has been considered an important parameter to assess the microbe impact on host energy requirements [8, 31, 47]. As observed the *Firmicutes* are increasing with the increase in roughage proportion while the *Bacteroidetes* population tends to decrease specifically during the dry fed diet. This leads to the increase in the F/B ratio, similar to the earlier reports where a shift from grain to fiber rich diets led to an increase in the ratio [31, 34, 48].

### Diet and Correlation between various Phyla

Microorganisms in natural environments are often involved in commensal or mutualistic interactions. Specifically, in the rumen significant interspecies cross feeding of intermediate products has been observed among the adherent and non adherent bacteria population [49]. Other interactions also include that between the carbohydrate fermenting bacteria which requires volatile fatty acids provided by the amino acid degrading ruminal bacteria [50]. In our study, we observed that the fibrolytic phyla *Fibrobacter* and *Firmicutes* positively correlated with the *Spirochaetes*. Studies have reported that co-culture of *F. succinogens* and a *Spirochaete* species *Treponema* enhanced fermentation of barley straw than a pure *F. succinogens* culture. Also, *Spirochaetes* decarboxylate the succinate formed by *Fibrobacter* into VFA which in turn serves as an important energy source to the host animal [51]. Results also revealed that *Bacteroidetes* positively correlated with the *Verrucomicrobia*, *Proteobacteria* and *Tenericutes* whereas negatively correlated with the cellulolytic *Fibrobacters* and *Firmicutes*. Previous studies on bovine rumen have reported *Bacteroidetes* to be negatively correlated with *Firmicutes* [31, 34].

### Functional diversity based on dietary changes

Functional metagenomics has paved the way in assessing potential metabolic activities of the rumen microbiome. The MG-RAST results suggest that protein metabolism had the highest hits followed by carbohydrate and both increased with increase in the roughage proportion. Initial colonization in ruminants is of the organisms possessing the enzymes to act on the side chains of complex plant polysaccharides followed by their complete degradation in the fluid fraction of rumen. Nitrogen metabolism was abundant in J3 followed by J1 and J2. At low protein diet, rumen bacteria are inclined to exploit an alternate source to fulfill the nitrogen requirement of host. Similar results were also observed in bovine rumen microbiome [38]. In fiber diet both leguminous and non leguminous plants are present and hence the bacteria can trap ammonia-N in the form of bacterial protein and their digestion supplies the needed amino acids to the host [37]. Significant difference in the solid and liquid fractions for nitrogen metabolism might indicate better interaction of fibrous legumes and nitrogen utilizing bacteria in the solid phase. Sulfur increased the efficiency of nitrogen utilization for animals consuming a fescue hay-based diet [52]. We also observed similar results in our study.

Further analysis at level 2 in protein metabolism, results stated that protein biosynthesis was dominant in all the samples, confirming that microbial protein synthesis acts as a major source of protein for ruminants [53]. In carbohydrate metabolism, the genes associated with one-carbon metabolism, involving methane metabolism category at level 3 were abundant. These were most abundant in the J1 treatment suggesting that methanogenic archaea tends to decrease with high roughage diet. This could also be on account of the subsequent decrease in pH [54]. The genes associated with CO_2_ fixation and central carbohydrate metabolism increased with roughage.

To assess the correlation among bacterial phyla, PCA was performed based on taxonomic and functional annotation. Overall, PCA according to the taxonomic and functional assignment of major categories showed that samples from dry and green roughage fed individuals of each treatment tended to cluster together, indicating that a similar set of functions were encoded in their metagenome.

### Diet impact on Rumen Metabolism

The estimation of VFA and various nitrogen moieties is necessary to track the microbial metabolism leading to the production of these compounds. VFA are extremely important as they endow more than 70 % of ruminants energy supply. Large proportion of the natural food proteins release ammonia, which is utilized by the rumen microbes for their growth and synthesis of microbial protein. VFA produced by microbial action in the rumen are absorbed by the epithelium and further carried to the liver via the blood circulatory system. This continuous absorption of VFA maintains the ruminal pH. In the present study, VFA concentration was highest in the low roughage fed individuals. There are similar reports with high VFA concentration in buffaloes [37, 55]. However, overall the concentration of VFA measured was comparatively lower compared to the earlier reports. The rumen microbiota converts ammonia into amino acids which is further accumulated as microbial protein. Total nitrogen was significantly different between the green and dry groups, indicating that the change in feed from green roughage to dry roughage led to significant change in the nitrogen content of rumen fluid. Also the N concentration was reduced from first treatment to the third. Similar differences in N content were observed in Mehsani buffalo fed on different diets and proportion of roughage [37]. Similar trend was observed for the NPN, soluble N and the ammonia N. The concentrations decreased with high roughage indicating enhanced consumption of nitrogen and higher microbial proteins synthesis.

### Dietary effect on volatile fatty acid (VFA) production

Rumen microbiome produces volatile fatty acids such as propionic acid, butyric acid and acetate by microbial fermentation, which in turn maintains the animal health and homeostasis. The VFAs are utilized further for milk and meat production contributing a major portion of the daily energy requirement of ruminants [56]. In our study, for propanoate production, out of the three plausible pathways, succinate pathway dominated. Similar to our results, several studies have reported that succinate pathway is considerably the most commonly used pathway by gut bacteria for propanoate production in mammals as well as humans [57, 58]. Propanoate production was well represented during all the three treatments, but maximum hits were observed during the first treatment. The observed results could be due to the high prevalence of *Bacteroidetes* during the first treatment [59]. Earlier reports on bovine rumen have stated that propanoate production increases in rumen at the expense of acetate and butyrate with increase in abundance of *Prevotella* sp. [60]. Similarly, butanoate production was also found to be well represented during the first treatment followed by second and third treatment, which is contradictory to that observed in Kankrej rumen where the production was lesser in equal proportion of roughage to concentrate compared to 75 % roughage diet [34]. Butanoate plays an important role in animal health by regulating the host physiology and as a signal molecule modulating cell properties like differentiation, proliferation and apoptosis [61].

### Dietary effect on Methanogenesis pathway

Methane production is an issue of immense concern worldwide for its role as a greenhouse gas [62]. Various sources of methane emission exist such as wetlands, energy sectors, biomass burning, landfills and ruminants [63] among which the enteric fermentation constitutes to be the largest source [64]. Alleviation of methane by studying the rumen archaea metabolism may contribute to devising strategies to decrease the greenhouse effect [65]. In this perspective, we determined the EGTs involved in methanogenesis and effect of each treatment on the same. Results reveal that an increase in roughage proportion reduced the EGTs involved in methanogenesis. The results are similar to previous studies on Gir cattle where similar decrease in methane production was observed with increase in roughage rich diet (Unpublished data).

### Diet based variation in CAZymes distribution

The potential to digest the coarse feed into metabolic energy in rumen is entirely attributed to its polysaccharide degrading enzymes. Rumen fluid due to its complex lignocellulosic degradation system is thus an excellent sample for mining CAZymes [38, 66]. In our study, oligosaccharide degrading enzymes were the most abundant of the total glycoside hydrolases. The higher occurrence of this category is justified due to their action on large number of oligosaccharides byproducts generated after initial breakdown of the complex polysaccharides like cellulose, hemicellulose, and pectin. Among the GHs, GH2 that corresponds for glucuronidase and GH3 corresponding for L-arabinofuranisidase, B-D-glucosidase and B-D-xylopyranosidase activities were the most abundant. The major source for GH3 is known to be *Prevotella* genus and hence we could observe that the proportion of GH3 encoding sequences decreased in the DL samples from J1 to J3 in correspondence to the decrease in *Prevotella* population as seen from the genus level classification of organisms encoding the GH genes [67]. Modifying the diet to higher roughage proportion has been found to decrease the amylase activity in most reports [37, 68]. Here we also observed similar trend whereby the GH13, 57 and 77 were reducing from J1 to J3. The presence of GH43 indicates microbial degradation of hemicellulose and xylose in rumen as this includes the α-L-arabinofuranosidases, endo-α-L-arabinanases and β-D-xylosidases. Variations in the abundance pattern of CAZy families among the green and dry roughage groups of the three treatments reveal the modulating microbial diversity with respect to feed as also observed from the abundance study on organisms involved in encoding these CAZymes.

## Conclusions

The findings of the present study reveal that dietary changes have a significant impact on rumen microbiome at taxonomic as well as functional levels. It also leads to change in the VFA production as well as on methanogenesis with increase in the roughage proportion of diet, subsequently having impact on the host as well as at the global level. Additionally, the findings also reveal that high roughage diet enhances the PEGs involved in microbial protein synthesis and reduces those involved in methane formation. The information generated may allow us to modulate the rumen microbiome by providing a formulated diet with high dry roughage proportion and limiting the amounts of elements in diet that are mandatory for enzymes involved in methanogenesis. This would ultimately improve livestock nutrient utilization efficiency for better agricultural yield and have a global impact on environment by controlling the emission of methane gas. The study enhances the overall understanding of Jaffrabadi buffalo rumen microbial ecology affected by diet, allowing us to explore the link between microbial metabolism and host physiology.

### Availability of Supporting Data

All the supporting data are included as additional files.

## Additional files

Additional file 1: Table S1. Detailed composition of the diet fed. (DOCX 11 kb) Additional file 2: Figure S1. Propanoate (VFA) production pathway (Abundance of enzymes during three treatments shown in parentheses). (JPG 365 kb) Additional file 3: Figure S2. Butanoate (VFA) production pathway (Abundance of enzymes during three treatments shown in parentheses). (JPG 691 kb) Additional file 4: Figure S3. Methanogenesis pathway (Abundance of enzymes during three different treatments shown in parentheses). (JPG 320 kb) Additional file 5: Figure S4. CAZyme family distribution throughout treatments (Animal replicates pooled). (JPG 644 kb)

## Abbreviations

CAZymes: Carbohydrate Active Enzymes
CAT: CAZyme Analysis Toolkit
CDS: Coding Sequence
D/G: Dry or green diet
EGTs: Environmental Gene Tags
GH: Glycosyl Hydrolase
J: Jaffrabadi
L/S: Liquid or solid fraction of rumen fluid
NPN: Non Protein Nitrogen
PCA: Principal Components Analysis
PEGs: Protein Encoding Genes
VFA: Volatile Fatty Acid
