# Supplementary material for: Effect of roughage on rumen microbiota composition in the efficient feed converter and sturdy Indian Jaffrabadi buffalo (Bubalus bubalis)
Source: BMC Genomics. 2015 Dec 29;16:1116. doi: 10.1186/s12864-015-2340-4 (PMC4696265; doi:10.1186/s12864-015-2340-4)
Supplement: Additional file 1: Table S1. — Detailed composition of the diet fed. (DOCX 11 kb) [file 12864_2015_2340_MOESM1_ESM.docx]

**Table S1:** Detailed composition of the diet fed

| **Component** | **Roughage Diet (%)** | | **Concentrate Diet (%)** |
| --- | --- | --- | --- |
|  | **Green** | **Dry** |  |
| Moisture | 82.26 | 6.31 | 5.74 |
| Crude Protein | 7.75 | 5.32 | 20.21 |
| Crude Fat | 0.94 | 1.44 | 1.87 |
| Crude Fibre | 32.85 | 31.00 | 12.57 |
| Acid Insoluble Ash | 3.36 | 2.53 | 3.84 |
