# Supplementary figures and images for: Effect of roughage on rumen microbiota composition in the efficient feed converter and sturdy Indian Jaffrabadi buffalo (Bubalus bubalis)
Source: BMC Genomics. 2015 Dec 29;16:1116. doi: 10.1186/s12864-015-2340-4 (PMC4696265; doi:10.1186/s12864-015-2340-4)

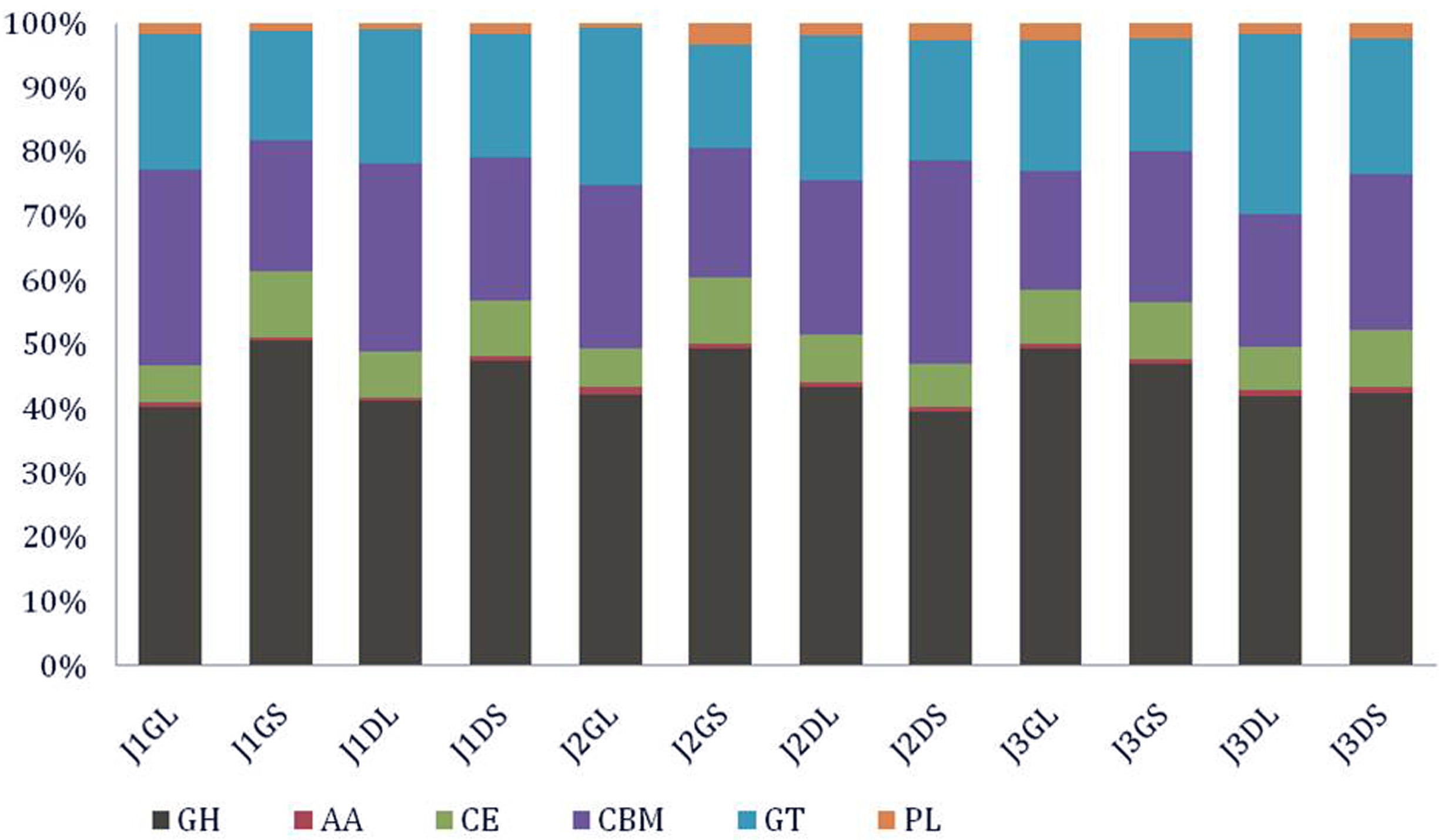

Supplement: Additional file 2: Figure S1. — Propanoate (VFA) production pathway (Abundance of enzymes during three treatments shown in parentheses). (JPG 365 kb) [file 12864_2015_2340_MOESM2_ESM.jpg]

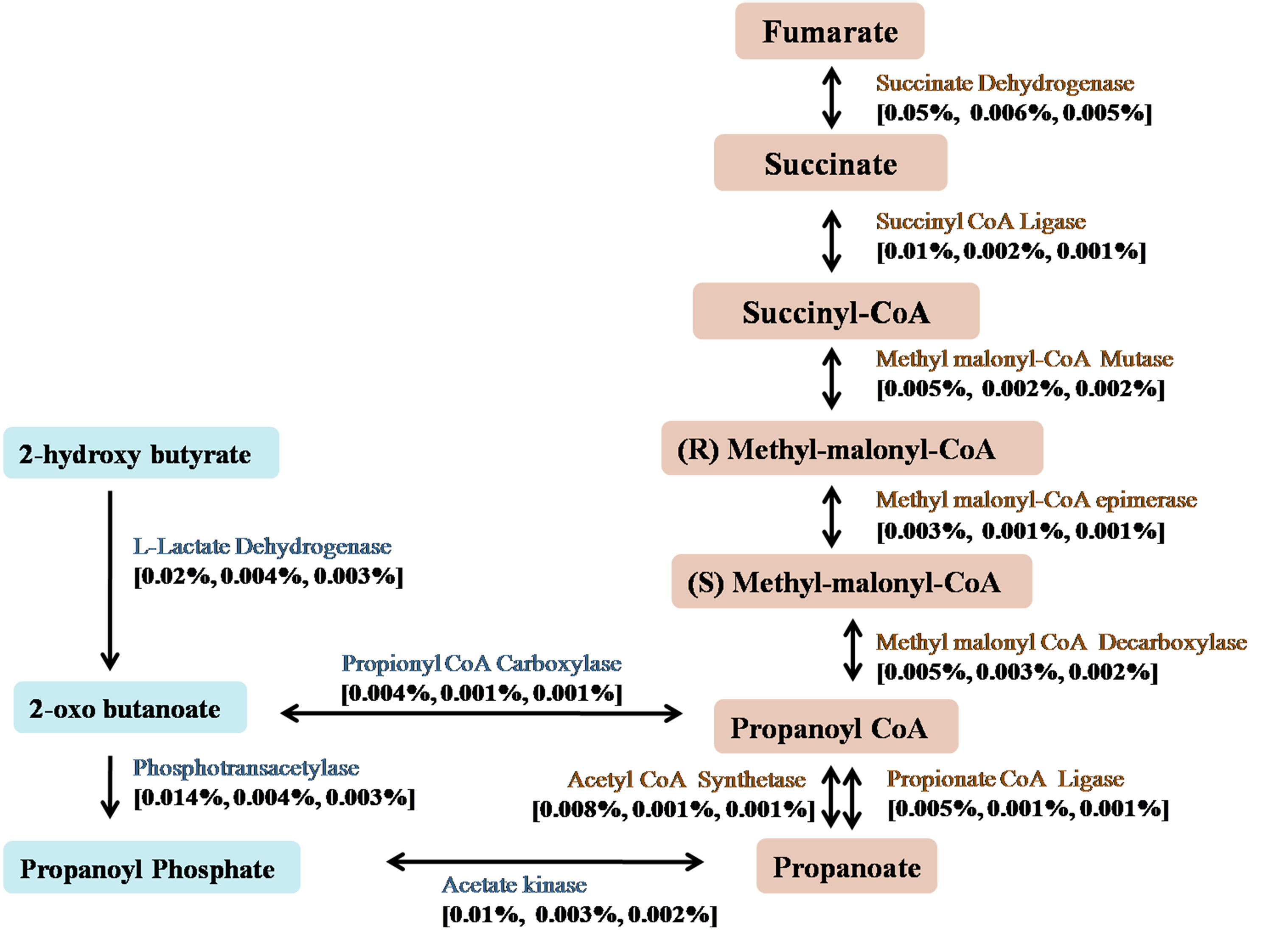

Supplement: Additional file 3: Figure S2. — Butanoate (VFA) production pathway (Abundance of enzymes during three treatments shown in parentheses). (JPG 691 kb) [file 12864_2015_2340_MOESM3_ESM.jpg]

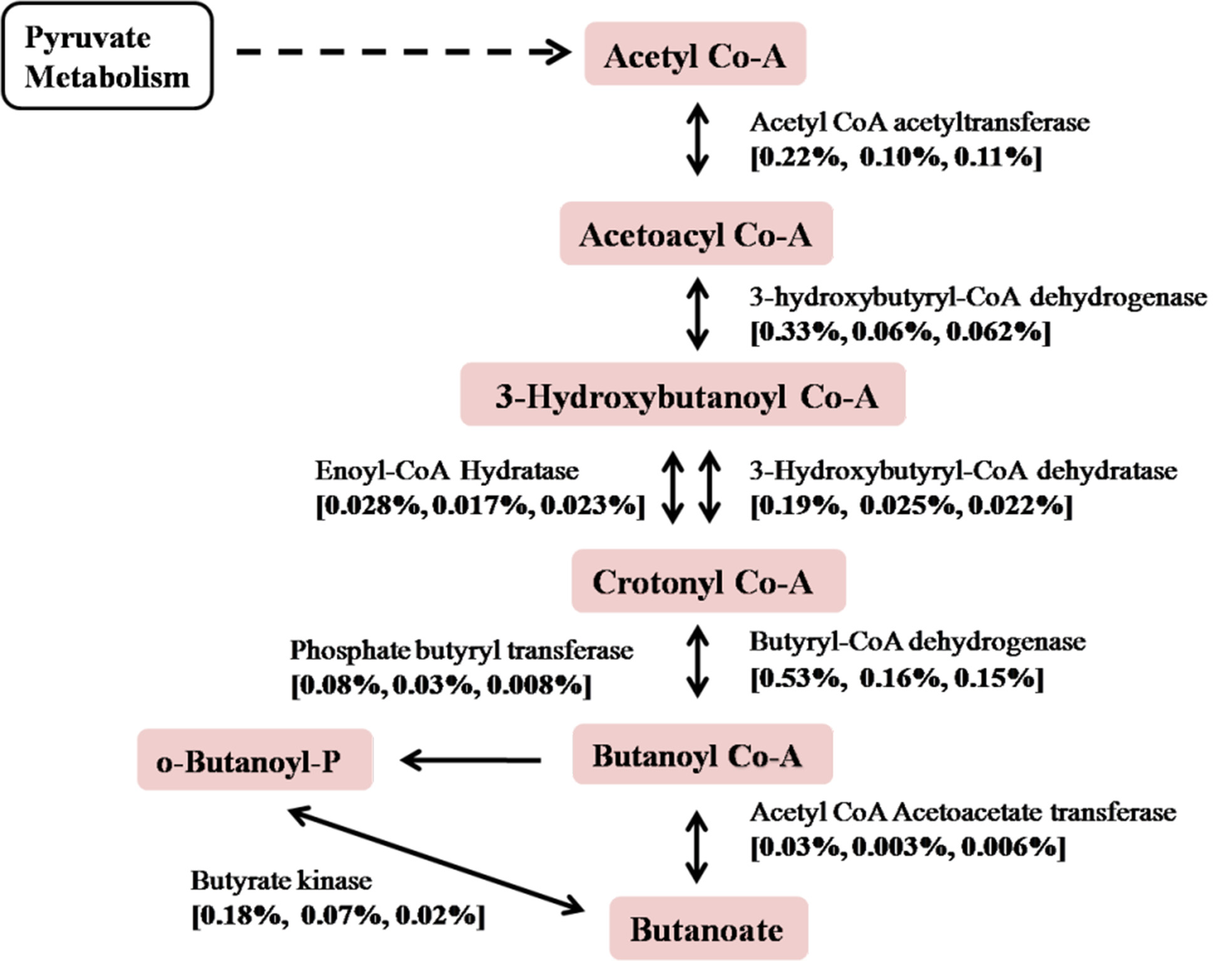

Supplement: Additional file 4: Figure S3. — Methanogenesis pathway (Abundance of enzymes during three different treatments shown in parentheses). (JPG 320 kb) [file 12864_2015_2340_MOESM4_ESM.jpg]

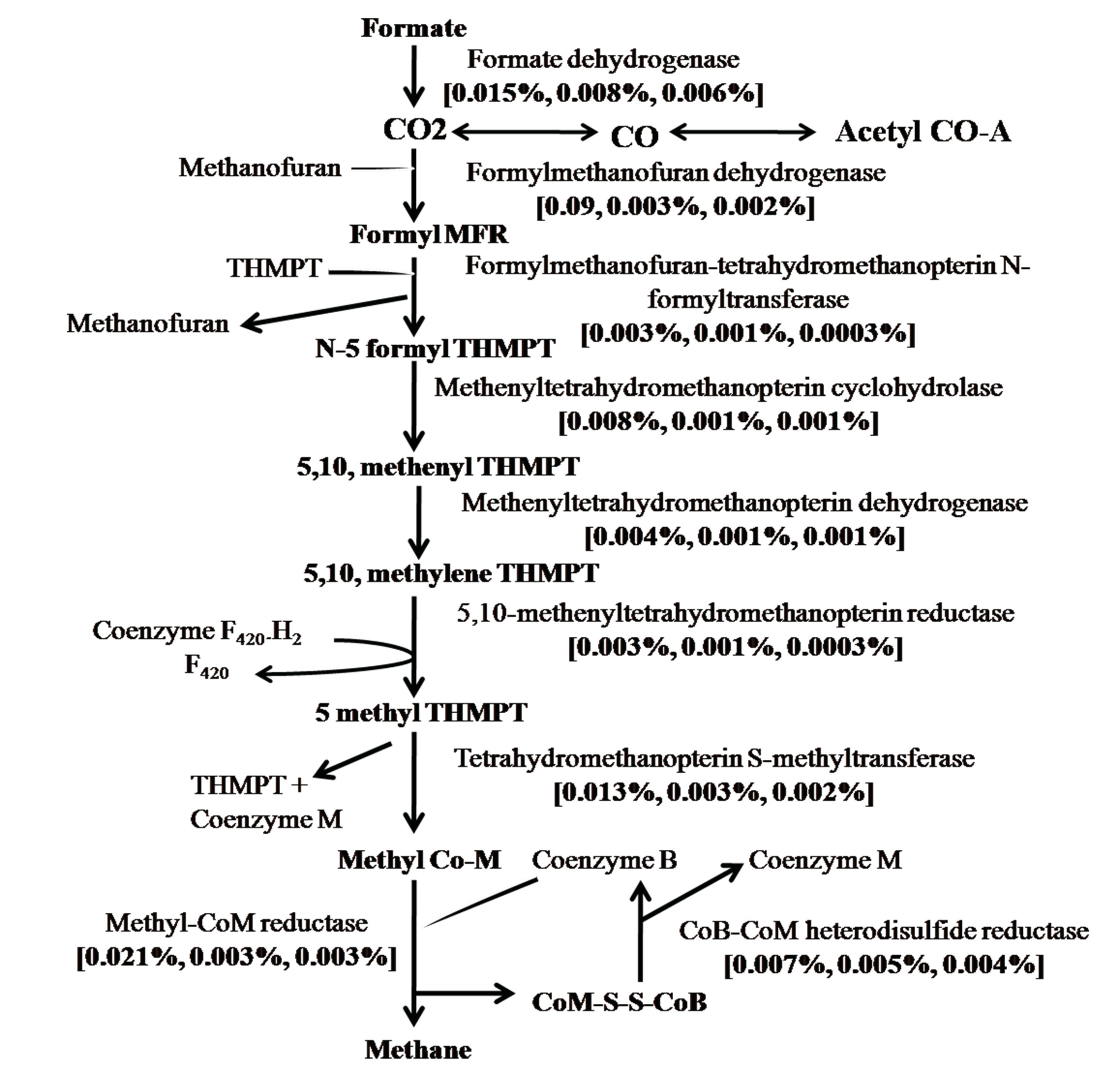

Supplement: Additional file 5: Figure S4. — CAZyme family distribution throughout treatments (Animal replicates pooled). (JPG 644 kb) [file 12864_2015_2340_MOESM5_ESM.jpg]
